# Occurrence of Harmful Algal Blooms in Freshwater Sources of Mindu and Nyumba ya Mungu Dams, Tanzania

**DOI:** 10.1155/2023/5532962

**Published:** 2023-10-16

**Authors:** Josephine J. Gobry, Hilda S. Bachwenkizi, Offoro N. Kimambo, Faustin N. Ngassapa, Kessy F. Kilulya

**Affiliations:** ^1^Department of Chemistry, College of Natural and Applied Science, University of Dar es Salaam, P.O. Box 35061, Dar es Salaam, Tanzania; ^2^Department of Water Resources, Water Institute, P.O. Box 35059, Dar es Salaam, Tanzania; ^3^Tanzania Agricultural Research Institute, Mikocheni, P.O. Box 6226, Dar es Salaam, Tanzania; ^4^Department of Geography & Environmental Studies, Sokoine University of Agriculture, Morogoro, Tanzania

## Abstract

Harmful algal blooms (HABs) pose a significant threat to aquatic ecosystems and human health due to the production of toxins. The identification and quantification of these toxins are crucial for water quality management decisions. This study used DNA analysis (PCR techniques) to identify toxin-producing strains and liquid-chromatography-tandem mass spectrometry (LC-MS/MS) to quantify microcystins in samples from Mindu and Nyumba ya Mungu Dams in Tanzania. The results showed that HABs were detected in both dams. The BLAST results revealed that the 16S gene sequences of uncultured samples were very similar to an *Antarctic cyanobacterium*, *Leptolyngbya sp*, *Anabaena sp*, and *Microcystis aeruginosa*. Sequences of the cultured samples were most similar to *Nodularia spumigena*, *Amazoninema brasiliense*, *Anabaena sp*, and *Microcystis aeruginosa*. Further analyses showed that the nucleotide sequence similarity of uncultured isolates from this study and those from the GenBank ranged from 85 to 100%. For cultured isolates from this study and others from the GenBank, nucleotide identity ranged from 81 to 100%. The molecular identification of *Microcystis aeruginosa* confirmed the presence of HABs in both Mindu and Nyumba ya Mungu Dams in Tanzania. At Mindu Dam, the mean concentrations (± standard deviation) of microcystin-LR, -RR, and -YR were 1.08 ± 0.749 ppm, 0.120 ± 0.0211 ppm, and 1.37 ± 0.862 ppm, respectively. Similarly, at Nyumba ya Mungu Dam, the concentrations of microcystin-LR, -RR, and -YR were 1.07 ± 0.499 ppm, 0.124 ± 0.0224 ppm, and 0.961 ± 0.408 ppm, respectively. This paper represents the first application of PCR and LC-MS/MS to study microcystins in small freshwater reservoirs in Tanzania. This study confirms the presence of toxin-producing strains of *Microcystis aeruginosa* in both dams and also provides evidence of the occurrence of microcystins from these strains. These findings contribute in improving the monitoring of HABs contamination and their potential impact on water quality in Tanzanian reservoirs.

## 1. Introduction

Globally, anthropogenic activities and climate change have negatively affected freshwater ecosystems, leading to a decline in water quality, biodiversity loss, and a reduction in ecosystem services [1]. Human pressure on land has been linked to increased eutrophication, which has deleterious effects on water bodies such as lakes and small reservoirs. Anthropogenic activities causing an increase in nutrients such as agricultural runoff and wastewater discharges contribute to eutrophication, which leads to an increase in HABs [1–4]. The growth of blue-green algae is facilitated by nitrogen and phosphorus inputs into waterbodies [5, 6], which has a significant negative impact on water quality and poses severe threats to aquatic life and public health [7]. The occurrence of HABs has been reported in different countries worldwide, including Tanzania [8–13]. Small inland waterbodies are especially vulnerable to nutrient enrichment and the eutrophication process due to their shallowness and relative isolation. They can develop strong physical-chemical gradients which make them susceptible to the growth of harmful blue-green algal blooms compared to large water bodies [14–18]. The Nyumba ya Mungu Dam and Mindu Dam are important man-made reservoirs in Tanzania but have been affected by eutrophication [19, 20].

Algal blooms are diverse and consist of a rapidly growing dominant group of organisms in freshwater environments [21, 22]. Many naturally occurring algal blooms in marine and freshwater habitats produce a range of toxins that can harm aquatic ecosystems and humans [23, 24]. Algal toxins produced by blue-green algae and their effect on human health are increasingly prevalent in freshwater systems worldwide [13, 23, 25]. The toxic bloom-forming species include the genera *Microcystis*, *Nodularia*, *Aphanizomenon*, *Anabaena*, *Cylindrospermopsis*, and *Oscillaroria* [23, 26–28]. Their toxins are classified according to their mode of action in vertebrates as hepatotoxins, cytotoxins, neurotoxins, dermatotoxins, and irritants [24, 27, 29]. Among all cyanotoxins, microcystin and nodularin are the most common hepatotoxins produced by a wide range of blue-green algae [29, 30]. The hepatotoxins have been reported to be potent protein phosphatase 1 (PP1) and 2A (PP2A) inhibitors. It has been revealed to have long-term cumulative toxic effects on potential tumour formation [31–33]. Local climate and weather strongly influence the occurrence, extent, and adaptations of blue-green algae to climatic fluctuations [12, 34].

Identification of HABs and their toxin levels is vital to water quality management decisions. Various methods have been employed for the detection of HABs. Microscopic analysis is a common method for identifying different algal species based on their morphological characteristics. However, this method can be time-consuming and labour-intensive, and it cannot distinguish between toxic and nontoxic algal strains [35–37]. Molecular techniques, such as DNA sequencing and polymerase chain reaction (PCR), are utilised to analyse the genetic material of collected samples. These methods can aid in identifying specific HAB species and strains by targeting and amplifying specific DNA markers [38–46]. PCR has proven to be a powerful method for detecting HAB species, as it can differentiate between toxic and nontoxic strains of *Microcystis* spp. [36]. Molecular techniques based on the presence or absence of genes necessary for toxin production have also been used to characterise toxic and nontoxic blue-green algae [47]. DNA isolation and gene amplification studies are commonly employed in molecular identification of species and phylogeny studies [48–50]. These methods enhance the accuracy and speed of organism identification [51].

Among the ten genes (*mcyA-J*) involved in microcystin production in blue-green algae, the *mcyE* of the microcystin synthetase enzyme plays a crucial role in synthesising all forms of microcystins [47, 52]. This area has been useful for detecting potential microcystin-producing blue-green algae in numerous algal blooms [53]. Using PCR, researchers have developed molecular-based techniques for identifying harmful *Microcystis* species in water [52, 54]. The method involves amplifying the microcystin synthetase (*mcy*) gene using universal and specific PCR primers [9, 55]. Partial sequences of the 16S rRNA gene determine the dominant species among blue-green algal communities in a particular habitat [47].

In terms of toxin quantification, several methods are utilised. Enzyme-linked immunosorbent assay (ELISA) utilises specific antibodies that bind to the target toxin, allowing for its detection and quantification. However, this method may have lower accuracy and sensitivity [35]. High-performance liquid chromatography (HPLC) is another commonly used technique, which separates and detects toxins based on their chemical properties such as molecular size and polarity. However, HPLC requires sophisticated instruments and involves complex analytical processes, which may limit its implementation [35]. Liquid chromatography-mass spectrometry (LC-MS) is gaining popularity due to its sensitivity [56–59]. It separates and detects toxins based on their mass and charge, enabling accurate quantification of multiple toxins in a sample [58].

Despite existing studies on the blue-green algae in freshwater reservoirs [12, 20, 60–64], there is limited information on the specific types of blue-green algae present, which can potentially cause HAB in Tanzania. In this study, the researchers used DNA analysis techniques (PCR and sequencing) to identify *Microcystis* strains and LC-MS/MS to quantify microcystins in the blooms and water samples from Mindu and Nyumba ya Mungu Dams. This study will help improve the monitoring of HABs contamination to ensure water safety for aquatic organisms, animals, and humans.

## 2. Materials and Methods

### 2.1. Study Area

**Figure 1:**
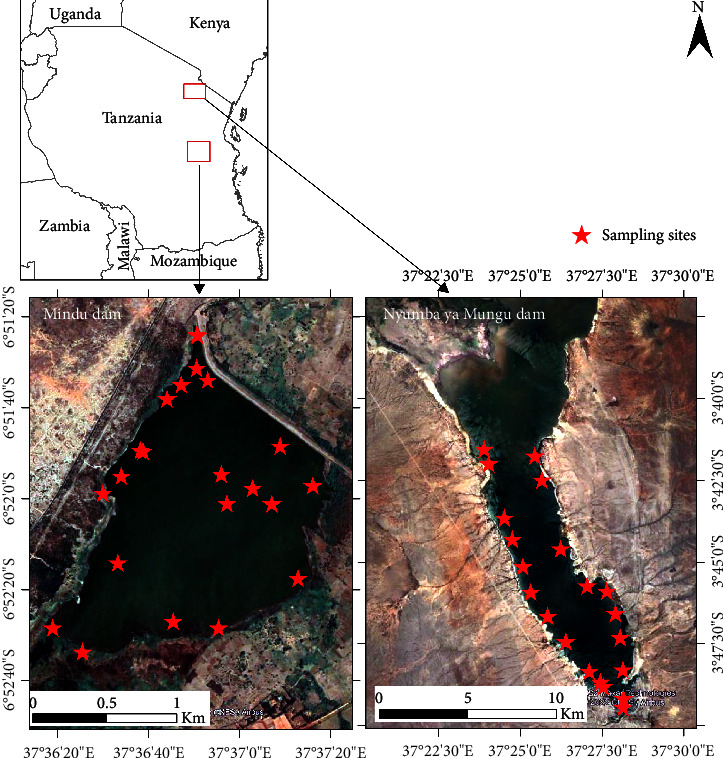
Map of Tanzania showing the study area and sampling points at Mindu Dam (right) and Nyumba ya Mungu Dam (left) (data source: own field data).

This study was conducted in two locations: the Mindu Dam in Morogoro and the Nyumba ya Mungu reservoir in Kilimanjaro (Figure 1). The reservoirs are used for agriculture, fishery, supply of raw potable water for domestic use, and recreational activities. Mindu Dam's area is approximately 3.8 km^2^, and that of its catchment is 303 km^2^ [65]. Nyumba ya Mungu Dam (140 km^2^) is the largest water body in the basin, and its catchment occupies a total land and water area of about 12,000 km^2^.

### 2.2. Sample Collection and Preparation

Water samples were randomly collected from Mindu and Nyumba ya Mungu Dams during the dry seasons from 2019 to 2021. In Mindu Dam, algal blooms were collected using polyethylene bags while in Nyumba ya Mungu Dam, water samples containing algal cells were collected by dipping sterile 500 mL amber glass sampling bottles into the surface water. All water samples for physical-chemical parameters from both dams were collected by using plastic bottles and preserved as per [66]. All samples were collected in triplicate from each of the sampling points. The bottles containing water samples and polyethylene bags containing algal blooms were packed into a cooler box containing ice blocks and transported to the Water Quality Laboratory at the Water Institute for further analysis. Other water quality variables measured to characterise the bloom conditions included temperature, dissolved oxygen (DO), electrical conductivity (EC), chlorophyll-a (Chl-a), nitrate (NO_3_), phosphate (PO_4_), pH, total dissolved solids (TDS), and turbidity were collected as per APHA [66].

Water samples from Nyumba ya Mungu Dam did not contain a high cell concentration by algal blooms as those from Mindu Dam. Therefore, they were first cultured for 21 days in laboratory environments using BG11 liquid media before DNA extraction. This allowed the algal cells to grow and multiply, making it easier to collect enough DNA for analysis. The BG11 liquid media were prepared using methods and conditions described by Kilulya and Msagati [67]. Five (5) millilitres of water containing algal cells were inoculated into the BG11 media. The flasks were incubated on a shaker at room temperature and in natural sunlight with 12-hour light and dark cycles for 21 days. The pH was maintained in the optimal range of 7.5 to 8.5 by adding sodium carbonate and citric acid buffers in controlled amounts to regulate the pH whenever it changed. The pH and temperature of the solution were measured daily to ensure that the conditions were optimal for algal growth.

### 2.3. Genomic DNA Extraction, PCR, and Sequencing

DNA was extracted using the cetyl trimethyl ammonium bromide (CTAB) method [68] from all uncultured samples (Mindu Dam) and cultured cells (Nyumba ya Mungu Dam). A Nanodrop 2000/2000C spectrophotometer (Thermo Scientific, Lagos Park, Porto Salvo) was used to measure the concentration and purity at 260 and 280 nm. The integrity of the isolated DNA in the samples was assessed by using agarose gel electrophoresis (1.2%) and staining with 0.1 g/ml ethidium bromide. The 16S rRNA genes of cultured water algal cells and uncultured algal bloom samples were first amplified from the genomic DNA using universal bacterial primers for the region. Universal 16S rRNA primers used were forward primer 27F: (5′-AGA GTT TGA TCC TGG CTC AG-3′) and reverse primer 1492R: (5′-TAC GCG CTA CCT TGT TAC GAC-3′) [69]. The *mcyE* gene region was amplified using the specific forward primer HEPF: (5′TTT GGG GTT AAC TTT TTT GGG CAT AGTC-3′) and reverse primer HEPR: (5′AAT TCT TGA GGC TGT AAA TCG GGT TT-3′) [53, 70].

The expected PCR fragment sizes were approximately 1.5 kb for the 16S rRNA gene and 472 bp for the microcystin *mcyE* gene. PCR reactions for all DNA samples were done using TEMpase Hot Start 2x Master Mix A as follows: 12.5 *μ*l of the master mix, 2 *μ*l of each forward and reverse primer (10 *μ*M), and 2 *μ*l of genomic DNA template in a final reaction volume of 25 *μ*l. The thermocycler conditions for TEMpase Hot Start 2x Master Mix A were as follows: initial denaturation at 95°C for 15 minutes followed by 35 cycles of 95°C for 30 seconds, 55°C for 45 seconds, 72°C for 1 minute, and finally extended at 72°C for 10 minutes. PCR conditions for specific microcystin genes were the same as for amplifying the 16S rRNA gene, except for the annealing time, which was shortened to 30 seconds. The amplicons were analysed on a 1.5% agarose gel and visualised using a UV light (BioDoc-ItTmImaging system, Cambridge, UK). After that, PCR products were sequenced at Inqaba Biotec (South Africa).

### 2.4. Sequence Analyses

The nucleotide sequences of the 16S rRNA and microcystin genes acquired from Sanger sequencing were checked for quality using the BioEdit program and aligned with other sequences obtained from GenBank (Table 1) using ClustaIW in MEGA X [71]. The maximum likelihood method based on the Tamura-Nei model and 1000 bootstrap replications were employed in the statistical analysis [72, 73]. The total number of positions in the final dataset for the 16S gene was 964, and for the mcyE gene, it was 472. The p-distance method was used to compute the evolutionary distances (expressed as numbers of base changes per site) [74]. The positions containing gaps and missing data were deleted entirely. The identities of nucleotide sequences were determined using the BioEdit sequence alignment editor [75]. The cleaned sequences were deposited in GenBank to get the accession numbers.

### 2.5. Quantification of Toxins in Microcystin-Producing Blue-Green Algae

Microcystin (MC) reference standards and prepared samples were analysed using the liquid-chromatography-tandem mass spectroscopy (LC-MS/MS) instrument. Freeze-dried algal bloom samples (0.5 g) were extracted twice with 70% methanol (v/v) by sonication for 10 minutes. The extracts were centrifuged at 4000 rpm for 10 minutes before solid-phase extraction (SPE). Supernatants were collected under vacuum at a flow rate of 1-2 mL/min and dried under a gentle nitrogen flow at 40°C. The residues were reconstituted with 0.5 mL of pure methanol. The samples were analysed by liquid-chromatography-electrospray ionization mass spectrometry (LC-ESI-MS/MS) using a Thermo Scientific™ Q Exactive Orbitrap instrument (Thermo Scientific, USA). Separation was achieved using a C18 Hypersil Gold column (100 mm × 2.1 mm, 1.9 *μ*m, ThermoScientific) kept at 35°C. The flow rate was set at 0.3 mL/min and sample injection volumes were 10 *μ*L. The mobile phases were water (mobile phase A) and acetonitrile (mobile phase B), both acidified with 0.1% formic acid (v/v). The gradient program started at 5% B (held for 2 minutes), increased to 100% B in 15 minutes, returned to initial conditions in 5 minutes, and equilibrated until 25 minutes. The ion source was operated in both positive and negative electrospray ionization modes for all experiments. Data were processed using Xcalibur version 4.1.31.9 software. Microcystins were quantified using the peak-area method described by Lawton and Edwards (2008). The retention time of standards was used to identify the peaks, and quantification was accomplished by using standard calibration curves.

### 2.6. Data Analysis

Data were tested for normality using the Shapiro–Wilks test. Upon confirmation of the normality assumption, a two-way ANOVA was conducted to test whether the different blue-green algae toxins differed significantly between the two study sites. A post hoc test (Tukey) was conducted to identify which pairs differed significantly. All the statistical analyses were conducted in the R STATS package. The results were considered significant at *p* < 0.05.

## 3. Results

Nine sequences of 16S rRNA gene sequences and one sequence of the *mcyE* gene were successfully deposited to GenBank. For Mindu Dam, the sequences were given codes: TZ: MO-MDS1 (Accession no. OP297379), TZ: MO-MDS2 (Accession no. OP297380), TZ: MO-MDS3 (Accession no. OP297381), and MO-MDS5 (Accession no. OP297382). The deposited nucleotide sequences from Nyumba ya Mungu Dam isolates were TZ: KL-NMS6 (Accession no. OP303798), TZ: KL-NMS7 (Accession no. OP303799), TZ: KL-NMS8 (Accession no. OP303800), TZ: KL-NMS9 (Accession no. OP303801), TZ: KL-NMS10 (Accession no. OP303802), and for the mcyE gene TZ:MO-MDS1gHep (Accession no. OP339494).

### 3.1. Genetic Variation and Phylogenetic Relationships (Mindu Dam)

The 16S rRNA identities of the Mindu Dam algal isolates were checked and confirmed through BLAST (https://blast.ncbi.nlm.nih.gov) analysis and are presented in Table 2. Four sequences obtained were deposited into the GenBank database. The four accession numbers for these sequences were OP297379, OP297380, OP297381, and OP297382; and they are similar to *Antarctic cyanobacterium* (Accession no. AY151723.1), *Leptolyngbya sp*. (Accession no. KT753322.1), *Anabaena sp*. (Accession no. HE975015.1), and *Microcystis aeruginosa* (Accession no. LC557463.1), respectively.

The identity of Tanzanian sequences from this study and other sequences from GenBank ranged from 85 to 100%. Four sequences from this study and six additional sequences from GenBank (representing various species of cyanobacteria and noncyanobacteria) were subjected to phylogenetic analysis. The accession numbers for sequences subjected to phylogenetic analysis of samples collected from Mindu Dam are shown in Table 2.

**Figure 2:**
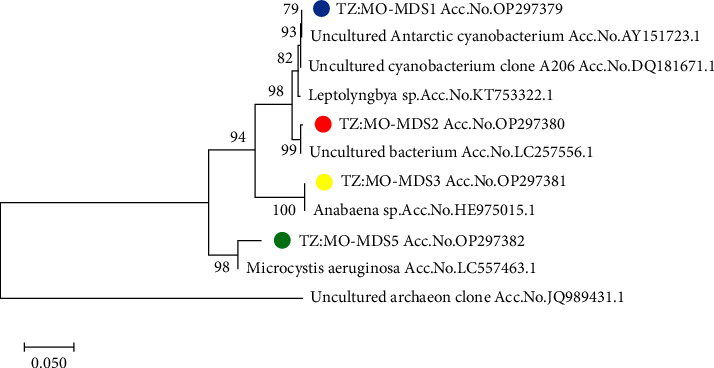
Maximum likelihood tree based on the 16S rRNA gene of uncultured algal bloom sequences (1500 bp) (sequences from other species were retrieved from GenBank). The coloured dots are the data from this study. The uncultured archaeon clone (JQ989431.1) was used as an out-group. 1000 bootstrap replications test was used to determine the nodes' robustness.

Four uncultured sequences from this study and six additional sequences from the database (representing various species of cyanobacteria and noncyanobacteria) were subjected to phylogenetic analysis. The results confirmed that the species from this study were related to four species from the database (Figure 2). The first group contains species from this study related to the *uncultured Antarctic cyanobacterium* with Accession no. AY151723.1; the second group contains species from this study related to the *uncultured bacterium* with Accession no. LC257556.1; and the third group contains species belonging to the genus *Anabaena* with Accession no. HE975015. The fourth group contains species from this study related to *Microcystis aeruginosa* with Accession no. LC557463.1.

### 3.2. Genetic Variation and Phylogenetic Relationships (Nyumba ya Mungu Dam)

**Figure 3:**
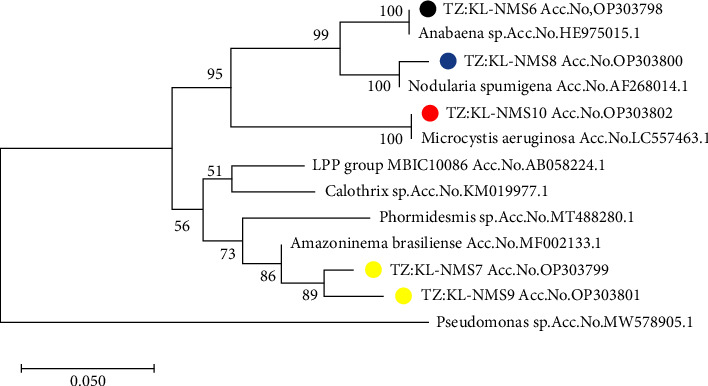
Maximum likelihood tree based on cultured water algal cell sequences of 16S rRNA gene (1500 bp). Sequences from other species were obtained from GenBank. The dots are the data of this study from Tanzania. *Pseudomonas* sp. (MW578905.1) was used as an out-group. Node robustness was assessed by performing 1000 bootstrap replications.

For the Nyumba ya Mungu Dam algal isolates, the nucleotide sequence identities of the 16S rRNA gene obtained through BLAST results are presented in Table 3. The five sequences were deposited in GenBank, and the accession numbers obtained for these 16S rRNA gene sequences are OP303798, OP303799, OP303798, OP303801, and OP303802. They were similar to *Anabaena* sp (Accession no. HE975015.1), *Amazoninema brasiliense* (Accession no. MF002133.1), *Nodularin spumigena* (Accession no. AF268014.1), *Amazoninema brasiliense* (Accession no. MF002133.1), and *Microcystis aeruginosa* (Accession no. MF002133.1), respectively. The nucleotide identities of Tanzanian species from this study and other species from the GenBank ranged from 81 to 100%. The phylogenetic tree of cultured 16S rRNA gene sequences showed that species are genetically diverse (Figure 3). Through phylogenetic analysis, the species were clustered into groups, i.e., *Amazoninema brasiliense*, *Nodularia spumigena*, *Microcystis aeruginosa*, and *Anabaena* spp with accession number listed in Table 3.

### 3.3. Genetic Variation and Phylogenetic Relationships

**Figure 4:**
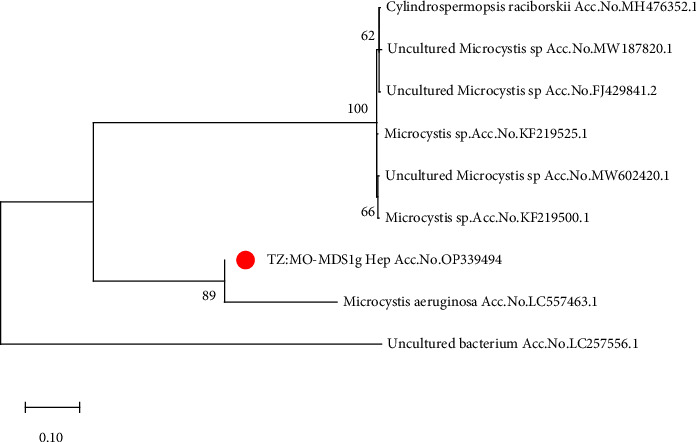
Maximum likelihood tree based on microcystin gene sequences (472 bp). GenBank was used to get sequences from other species. The red dot is the data of this study from Tanzania. The uncultured bacterium sp. (Accession no. LC257556.1), indicated with a black colour, was used as an out-group. Node robustness was assessed by performing 1000 bootstrap replications.

The nucleotide sequence of the microcystin *mcyE* gene of TZ: MO-MDS1g_Hep (Accession no. OP339494) showed a 96.77% identity to the sequence from *Microcystis aeruginosa* (Accession no. LC557463.1) based on BLAST results and GenBank sequences. A phylogenetic tree of the microcystin gene sequences confirmed that they were closely related to *Microcystis aeruginosa* (Accession no LC557463.1) and distantly related to *Cylindrospermopsis raciborskii* (Accession no. MH476352.1 (Figure 4).

### 3.4. Toxins in Microcystin-Producing Blue-Green Algae

Table 4 presents the summary statistics for different blue-green algal toxins detected at Nyumba ya Mungu and Mindu Dams. At Mindu Dam, the means and standard deviations for microcystin-LR, -RR, and -YR were 1.08 ± 0.749 ppm, 0.120 ± 0.0211 ppm, and 1.37 ± 0.862 ppm, respectively. At Nyumba ya Mungu, the means and standard deviations for microcystin-LR, -RR, and -YR were 1.07 ± 0.499 ppm, 0.124 ± 0.0224 ppm, and 0.961 ± 0.408 ppm, respectively. ANOVA test results revealed a significant difference in concentration among the three toxins (*F* = 16.26, df (2), *p* < 0.001). However, toxin levels between the two sites were statistically the same (*p* > 0.05), and there was insignificant interaction between toxin type and location (*F* = 0.81, *p*=0.449). The results from a post hoc test indicated that the microcystin-RR concentration was significantly lower than microcystin-LR and microcystin-YR (*p* < 0.001). In contrast, the concentrations of microcystin-YR and microcystin-LR were not significantly different (*p*=0.729).

### 3.5. Water Quality Variables and Their Influence on Algal Bloom

The results of various water quality parameters collected from the two study sites are presented in Table 5. The analysis revealed statistically significant spatial variations between the two sites (*p* < 0.05) for all parameters except total phosphates (*p* > 0.05). Nyumba ya Mungu Dam exhibited significantly higher temperature values, dissolved oxygen (DO), and electrical conductivity (EC) than the Mindu Dam. Conversely, the Mindu Dam displayed significantly higher levels of chlorophyll-a (Chl-a), nitrate (NO_3_), pH, and turbidity than the Nyumba ya Mungu Dam.

### 3.6. Correlations among Water Quality Parameters

**Figure 5:**
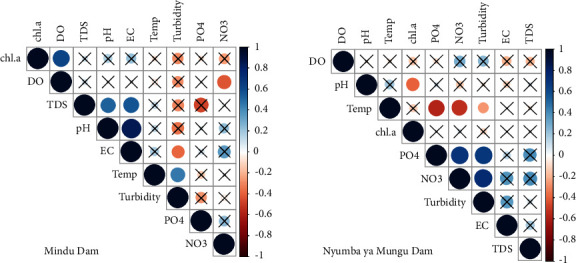
Correlations among the water quality parameters. Positive correlations are represented in blue, while negative correlations are shown in red. The colour's intensity and the circles size are proportional to the correlation coefficients. Crosses denote correlations considered statistically insignificant (*p* value >0.05).

Correlations among the water quality parameters were analysed using Spearman's correlation test. The results (Figure 5) revealed that the nature of the correlations varied between the two sites. At Mindu Dam, significant positive correlations (*p* < 0.05) were observed between Chl-a and DO, TDS and pH, and EC and pH, as well as temperature and turbidity. A significant negative correlation was observed between EC and turbidity, as well as DO and NO_3_. On the other hand, at Nyumba ya Mungu Dam, a statistically significant positive correlation was observed between PO_4_ and NO_3_, PO_4_ and turbidity, as well as NO_3_ and turbidity (*p* < 0.05). A significant negative correlation was found between Chl-a and pH, temperature and PO4, and NO_3_ and turbidity.

## 4. Discussion

This study aimed to identify toxin-forming blue-green algae and quantify the levels of toxins, specifically focusing on *Microcystis aeruginosa*. Our findings confirm the presence of *Microcystis* species in both the Mindu and Nyumba ya Mungu Dams, indicating the occurrence of toxin-forming blue-green algae in these environments. The maximum likelihood tree derived from the analysis of the 16S rRNA uncultured algal bloom sequences from Mindu Dam exhibited distinct and accurately identified species (Figure 2). All species in the tree were categorised as blue-green algae, except for TZ: MO-MDS2 (Accession no. OP297380), which showed a close relation to an *uncultured bacterium species*. In the phylogenetic tree, TZ: MO-MDS5 (Accession no. OP297382) clustered phylogenetically with *Microcystis aeruginosa* (Accession no. LC557463.1). Another species with high sequence identity was TZ: MO-MDS1 (Accession no. OP297379), which demonstrated a 100% phylogenetic relation to an *uncultured Antarctica cyanobacterium* and 99% to *Leptolyngbya sp*. The isolate TZ: MO-MDS3 (Accession no. OP297381) exhibited a 100% relation to Anabaena *spp*. These findings are consistent with those of Kimambo et al. [2], who characterised common species of blue-green algal blooms.

In Nyumba ya Mungu Dam, no species closely related to noncyanobacteria were identified (Figure 3). The isolates TZ: KL-NMS7 (OP303799) and TZ: KL-NMS9 (OP303801) were closely related to *Amazoninema brasiliense* (Accession no. MF002133.1), previously reported by Yadav et al. [76]. The isolate TZ: KL-NMS6 (Accession no. OP303798) was closely related to Anabaena sp. (Accession no. HE975015.1), which aligns with the findings of Kimambo et al. [2] who also reported the presence of this species in Tanzanian freshwater. TZ: KL-NMS8 (Accession no. OP303800) exhibited a close relation to *Nodularia spumigena* (Accession no. AF268014.1), and TZ: KL-NMS10 (Accession no. OP303802) was closely related to *Microcystis aeruginosa* (Accession no. LC557463.1) found in uncultured algal blooms. These findings agree with those of Mchau et al. [63], who reported the presence of microcystins in Lake Victoria, Tanzania.

A polymerase chain reaction (PCR) test was used to screen for the presence of genes associated with the production of toxins. The mcyE gene was detected, which is the gene responsible for the production of toxic genes. The use of gene-specific primers confirmed the presence of toxic genes in the dams. The phylogenetic trees for the amplified microcystin gene (Figure 4) indicate a close relationship with *Microcystis aeruginosa* (Accession no. LC557463.1). Chemical analysis further confirmed that toxic genes were present in the dams. These findings agree with those of Benredjem et al. [70]. The presence of different species of blue-green algae (cyanobacteria), noncyanobacteria, and *Microcystis* species in this study reveals high genetic diversity, as these species cluster with different species reported in previous studies by Kimambo et al. [2]; Yuan et al. [35]; Yadav et al. [76]; and Karan et al. [69].

We also quantified the blue-green algal toxins microcystin-LR, -RR, and -YR during this study. The concentrations of all toxins were within the WHO advisory limits for safe recreational water environments [77]. The WHO's concentration limits for microcystin-LR, -RR, and -YR in safe recreational water environments are 60 (25–125), 70, and 300–600 *μ*g/kg, respectively. WHO requires a maximum acceptable concentration of 1 *μ*g/kg for the blue-green algal toxin microcystin-LR for drinking water [78].

Comparing our results with previous studies, we identified consistencies and differences. Kimambo et al. [2] also reported the presence of *Microcystis* species in Tanzanian freshwater, supporting our findings. Furthermore, Mchau et al. [63] quantified Microcystins in Lake Victoria, Tanzania, further supporting our results. These consistencies suggest a shared occurrence of *Microcystis* species and their toxin production in various Tanzanian freshwater bodies.

A study of water quality in two dams found that there were significant differences between the two sites. The Mindu Dam had higher levels of chlorophyll-a, DO, TDS, EC, temperature, and turbidity. The Nyumba ya Mungu Dam had lower levels of chlorophyll-a and only showed weak positive associations with PO_4_, NO_3_, and turbidity. These findings highlight the complex interplay between chlorophyll-a and other water quality variables. The prevailing environmental conditions in the two reservoirs are likely to play a significant role in supporting and enhancing the production of toxins through algal blooms. Our study did not find a clear link between chlorophyll-a and nutrient levels. This could be due to the timing of our sampling. The findings are inline with those of Pérez-Ruzafa et al. [79] that reported low correlation of chlorophyll-a to nutrients in small areas and over short periods time. In addition, it is important to note that the growth of chlorophyll-a is not solely determined by nitrate and phosphate levels. Other factors, such as light availability [80] and various micronutrients [81], exert considerable influence on the dynamics of chlorophyll-a. These nuanced factors were regrettably not taken into account within the scope of this particular study. The study provides valuable insights into the connections between water quality variables and algal blooms, but it has some limitations. It is important to note that the complex nature of natural systems makes it difficult to isolate individual factors that contribute to bloom dynamics. The study did not delve into the precise mechanisms that trigger toxin production by algae. In addition, the correlations that were found do not necessarily prove causation. Additional research, including controlled experiments, is needed to uncover the exact interplay between water quality variables and bloom dynamics.

Despite the stated limitations, our results contribute to understanding the genetic diversity and toxin production of cyanobacteria in Tanzanian water bodies. The findings can guide future studies on the occurrence, distribution, and potential risks associated with toxin-forming cyanobacteria in freshwater ecosystems. The practical applications of this study are significant, as they underscore the importance of regular monitoring and assessment of water bodies for the presence of toxin-forming blue-green algae. This study highlights the need to implement effective water quality management strategies and public health protection, particularly in areas with prevalent blue-green algal blooms. Furthermore, our findings can provide valuable insights for crafting focused mitigation and control strategies aimed at minimizing the risks linked to blue-green algal toxins. This can be achieved through the formulation of site-specific management plans that comprehensively address the factors that contribute to both bloom formation and toxin production.

Overall, our study extends previous research findings by providing molecular identification of toxin-forming blue-green algae and quantifying toxin levels in selected strains, with a specific focus on *Microcystis aeruginosa*. By comparing our results with other studies, we gain a broader understanding of blue-green algal diversity, toxin production, and potential variations across different water bodies. However, further investigations are warranted to explore additional genetic markers and conduct comprehensive assessments to enhance our understanding of blue-green algal dynamics and their associated toxins in diverse aquatic environments.

## 5. Conclusion

In conclusion, this study provides valuable insights into the presence and genetic diversity of blue-green algae (cyanobacteria) in Tanzanian waterbodies, shedding light on their potential risks and implications for water quality management and public health protection. By confirming the occurrence of *Microcystis* species and their toxin production in the Mindu and Nyumba ya Mungu Dams, we contribute to the understanding of blue-green algal dynamics in these environments. The consistent findings with previous studies confirm the robustness of our results and support the shared occurrence of *Microcystis* species in Tanzanian freshwater.

Identifying specific genetic markers and quantifying blue-green algal toxins highlight the importance of regular monitoring and assessment to mitigate the risks associated with blue-green algal blooms. The study emphasises the need for implementing effective strategies for water quality management and underscores the significance of targeted mitigation measures to safeguard public health. The findings contribute to a broader understanding of blue-green algal dynamics and their associated toxins in Tanzanian water bodies and freshwater ecosystems worldwide. This study is a stepping stone for future research, encouraging further investigations into additional genetic markers and comprehensive assessments to enhance our understanding of blue-green algal ecology and their potential impacts.

## Figures and Tables

**Table 1:** The list of taxa and their GenBank accession numbers used for phylogenetic analysis of the 10 sequences obtained from this study.

Gene region			
16s rRNA gene		*mcyE* gene	
Species names	Accession numbers	Species names	Accession no.
*Antarctic cyanobacterium*	AY151723.1	*Cylindrospermopsis raciborskii*	MH476352.1
*Leptolyngbya sp*	KT753322.1	Uncultured bacterium sp.	LC257556.1
*Anabaena sp*	HE975015.1	*Pseudomonas sp.*	MW578905.1
*Microcystis aeruginosa*	LC557463.1	*Microcystis sp.*	KF219525.1
Uncultured cyanobacterium clone A206	DQ181671.1	*Microcystis sp.*	KF219500.1
Uncultured bacterium	LC257556.1	Uncultured *Microcystis sp*	MW602420.1
*Amazoninema brasiliense*	MF002133.1	Uncultured_*Microcystis sp*	FJ429841.2
*Nodularin spumigena*	AF268014.1	Uncultured *Microcystis sp*	MW187820.1
LPP-group MBIC10086	AB058224.1	*Microcystis aeruginosa*	LC557463.1
*Phormidesmis sp.*	MT488280.1		
*Calothrix sp*	KM019977.1		

**Table 2:** Identities of the 16S rRNA gene for blue-green algae obtained from Mindu Dam samples.

Sequences from this study					Sequences from the GeneBank		
Type of samples^1^	The location where samples were collected^2^	Region	Assigned isolate names	Accession numbers^3^	Closest related species in GenBank	Query cover (%)	Identities (%)^4^
AB	MD	Morogoro	TZ: MO-MDS1	OP297379	*Antarctic cyanobacterium* (Accession no. AY151723.1) *Leptolyngbya sp*	100	100
					(Accession no. KT753322.1)		
AB	MD	Morogoro	TZ: MO-MDS2	OP297380	Uncultured bacterium (Accession no. LC257556.1)	100	99
AB	MD	Morogoro	TZ: MO-MDS3	OP297381	*Anabaena sp*. (Accession no. HE975015.1)	100	100
AB	MD	Morogoro	TZ: MO-MDS5	OP297382	*Microcystis aeruginosa* (Accession no. LC557463.1)	100	98

^1^AB: algal bloom; ^2^MD: Mindu Dam; ^3^refers to accession numbers issued by NCBI following the submission of the nucleotide sequences; ^4^the automatically generated identities obtained using BLAST in GenBank hosted by NCBI.

**Table 3:** Identities of partial nucleotides sequence of blue-green algae obtained from Nyumba ya Mungu Dam samples.

Sequences from this study					Sequences from the GeneBank		
Type of samples^1^	The location where samples were collected^2^	Region	Assigned isolate names	Accession numbers^3^	Close related species in GenBank	Query cover (%)	Identity (%)^4^
WA	NYM	Kilimanjaro	TZ: KL-NMS6	OP303798	*Anabaena sp.* (Accession no. HE975015.1)	100	100
WA	NYM	Kilimanjaro	TZ: KL-NMS7	OP303799	*Amazoninema brasiliense* (Accession no. MF002133.1)	99	97
WA	NYM	Kilimanjaro	TZ: KL-NMS8	OP303800	*Nodularin spumigena* (Accession no. AF268014.1)	99	94
WA	NYM	Kilimanjaro	TZ: KL-NMS9	OP303801	*Amazoninema brasiliense* (Accession no. MF002133.1)	99	97
WA	NYM	Kilimanjaro	TZ: KL-NMS10	OP303802	*Microcystis aeruginosa* (Accession no. LC557463.1)	100	100

^1^WA = water algal; ^2^NYM = Nyumba ya Mungu; ^3^refers to accession numbers issued by NCBI following the submission of the nucleotide sequences; ^4^the automatically generated identities during the BLAST process in the GenBank.

**Table 4:** Summary statistics for different types of blue-green algae toxins at Nyumba ya Mungu and Mindu Dam.

Toxins	Mindu Dam (*N* = 38)	Nyumba ya Mungu Dam (*N* = 24)
	Mean ± SD	Mean ± SD
Microcystin-LR	1.08 ± 0.749^a^	1.07 ± 0.499^a^
Microcystin-RR	0.120 ± 0.021^b^	0.124 ± 0.022^b^
Microcystin-YR	1.37 ± 0.862^a^	0.961 ± 0.408^a^

Columns not sharing the same letter in superscript are statistically different (*p*<0.05).

**Table 5:** Descriptive statistics for water quality parameters in Nyumba ya Mungu and Mindu Dams.

Parameters	Site		
	Nyumba ya Mungu Dam (*N* = 21)	Mindu Dam (*N* = 21)	*p* value
Chl-a	6.37 ± 4.66^a^	13.2 ± 9.07^b^	0.008
PO_4_	0.658 ± 0.154^a^	0.583 ± 0.196^a^	0.2084
NO_3_	0.495 ± 0.624^a^	1.38 ± 0.912^b^	<0.001
pH	9.42 ± 0.0707^a^	11.6 ± 17.6^b^	<0.001
Temp	25.2 ± 1.38^a^	23.5 ± 1.60^b^	0.002
DO	12.8 ± 3.00^a^	5.30 ± 0.870^b^	<0.001
Turbidity	6.83 ± 3.84^a^	18.6 ± 2.73^b^	<0.001
EC	673 ± 12.0^a^	225 ± 3.97^b^	<0.001
TDS	363 ± 15.5^a^	125 ± 3.06^b^	<0.001

Rows sharing the same letter within each parameter are not significantly different from each other (*p* > 0.05).

## Data Availability

The data used to support the findings of this study are available from the corresponding author upon request.
